# What motivates patients and caregivers to engage in health research and how engagement affects their lives: Qualitative survey findings

**DOI:** 10.1111/hex.12979

**Published:** 2019-12-04

**Authors:** Rachel Hemphill, Laura P. Forsythe, Andrea L. Heckert, Andrew Amolegbe, Maureen Maurer, Kristin L. Carman, Rikki Mangrum, Lisa Stewart, Ninma Fearon, Laura Esmail

**Affiliations:** ^1^ Patient‐Centered Outcomes Research Institute Washington DC USA; ^2^ American Institutes for Research Chapel Hill NC USA

**Keywords:** comparative effectiveness research, motivation, patient engagement, patient participation, patient‐centered research, stakeholder participation

## Abstract

**Background:**

US research organizations increasingly are supporting patient and stakeholder engagement in health research with a goal of producing more useful, relevant and patient‐centered evidence better aligned with real‐world clinical needs. The Patient‐Centered Outcomes Research Institute (PCORI) engages patients, family caregivers and other health‐care stakeholders, including clinicians, payers and policymakers, as active partners in prioritizing, designing, conducting and disseminating research as a key strategy to produce useful evidence for health‐care decision making.

**Objective:**

To inform effective engagement practices and policies, we sought to understand what motivates patients and caregivers to engage as partners on PCORI‐funded research projects and how such engagement changed their lives.

**Methods:**

We conducted thematic analysis of open‐ended survey responses from 255 patients, family caregivers and individuals from advocacy and community‐based organizations who engaged as partners on 139 PCORI‐funded research projects focusing on a range of health conditions.

**Results:**

Partners' motivations for engaging in research were oriented primarily towards benefiting others, including a desire to improve patients' lives and to support effective health‐care interventions. In addition to feeling they made a positive difference, many partners reported direct benefits from engagement, such as new relationships and improved health habits.

**Discussion and Conclusions:**

By identifying patient and caregiver motivations for engaging in research partnerships and what they get out of the experience, our study may help research teams and organizations attract partners and foster more satisfying and sustainable partnerships. Our findings also add to evidence that engagement benefits the people involved as partners, strengthening the case for more widespread engagement.

## 1 INTRODUCTION

Patients, family caregivers and organizations that represent them are important consumers of health research. Meaningful involvement of patients and caregivers in health research has long been identified by participatory research advocates as necessary to ensure that research and evidence are relevant, useful and trusted by individuals and communities.^1^, ^2^ Such involvement, also known as engagement in research, occurs when patients and caregivers are active partners in prioritizing, designing, conducting and disseminating research. In the last decade, policies and initiatives promoting patient engagement in health care broadly and in research specifically have gained prominence in the United States as a key strategy to make the health‐care system more patient‐centered and efficient and to achieve better health outcomes for individuals (eg^3^, ^4^, ^5^). A growing body of literature suggests that patient engagement can improve evidence for decision making by ensuring that research questions and outcomes studied are relevant to patients' needs, that studies successfully enrol and retain participants, and that findings are shared with those who need them (eg^6^, ^7^, ^8^, ^9^, ^10^).

While a promising strategy to improve research evidence, engaging patients and other stakeholders in research partnerships requires resources and effort.^8^, ^11^, ^12^, ^13^ Researchers and partners may face challenges related to building relationships and communicating, including ensuring that partners feel heard and valued, managing expectations about project progress or roles, and maintaining consistent partner participation.^11^, ^13^, ^14^, ^15^ Ultimately, fostering mutually beneficial and sustainable engagement on a larger scale will require more effective approaches for attracting research partners, engaging them in the research process and maintaining relationships.

Understanding the experiences of patients and caregivers who engage as research partners, particularly their motivations and perceptions of how engaging affects their lives, can inform strategies for developing and sustaining satisfying research partnerships. Yet, the current engagement literature focuses mostly on the effects of engagement on research processes and outcomes, with less attention paid to the people engaged as research partners. A UK study found that patients and caregivers describe several altruistic and personal motivations for engaging in research, such as making a difference in patient care, giving something back to the National Health Service and gaining a better understanding of health problems.^16^ Other research, also conducted mainly in the UK, found that patients and caregivers report personal benefits of engaging as research partners, including feeling valued and empowered and gaining new skills and better knowledge of research.^11^, ^16^, ^17^, ^18^, ^19^ Less commonly, patients report negative effects of engaging, such as feeling undervalued by researchers, feeling burdened by demands of the role and not knowing if their input makes a difference.^11^, ^16^, ^19^ Patient and caregiver motivations for engaging in research and their perceptions of how engagement affects their lives have not been explored on a large or systematic scale in the United States, where engagement is a newer practice and some aspects of health research and care delivery are unique.

The creation of the Patient‐Centered Outcomes Research Institute (PCORI) provides an opportunity to study engagement in research on a larger scale than has been done previously and expand understanding of engagement in the United States. Authorized by Congress in 2010 to fund comparative clinical effectiveness research (CER),^20^ PCORI requires engagement of patients, family caregivers and other health‐care stakeholders, such as clinicians, health systems, payers and policymakers, in its funded research. PCORI is unique among US health research funders in the scale and scope of its engagement requirements and systematic efforts to collect and analyse information about engagement. Now that hundreds of PCORI‐funded research projects are underway or completed, we can expand current knowledge about engagement by learning directly from PCORI research teams. This study focuses on identifying and describing partners' motivations for engaging in research and how engagement changed their lives. We analysed data collected from a sample of patients, family caregivers and individuals from organizations that represent patients and caregivers who engaged as partners on PCORI‐funded research projects.

## 2 MATERIALS AND METHODS

This exploratory qualitative study is part of a larger mixed‐methods study using survey data to examine experiences of patients and other stakeholders engaged as partners on PCORI research projects. MaGil Institutional Review Board (now Advarra) approved this research.

### 2.1 Patient and stakeholder involvement

PCORI's Advisory Panel on Patient Engagement, which recommends how to ensure patient‐centeredness in PCORI's work, guided this study from inception. The panel is comprised primarily of patients and caregivers, along with researchers, clinicians and other stakeholders. Advisory panellists informed this study's research questions, data collection tool, analytic approach and interpretation of findings.

### 2.2 Data collection

PCORI collected data between March 2016 and July 2018 from patients and other stakeholders engaged as partners on PCORI‐funded research projects. As part of annual reporting requirements, 303 principal investigators were asked to nominate up to 10 stakeholder partners per project to share their experiences by answering questions using the Ways of Engaging‐Engagement Activity Tool (WE‐ENACT). Approximately 70%, or 211 investigators, nominated at least one partner. Nominated partners received an email invitation to complete the WE‐ENACT, with up to three email reminders. The WE‐ENACT was administered via a web‐based survey platform, or by phone if desired, and participation was voluntary. Partners did not receive payment or other forms of compensation for participating.

PCORI staff developed and refined the WE‐ENACT based on past data collection efforts,^12^ PCORI's Evaluation Framework,^21^ PCORI's conceptual model of PCOR,^4^ input from PCORI's Advisory Panel on Patient Engagement and the published literature. The survey included closed‐ and open‐ended questions about partners' characteristics and experiences engaging in the PCORI project (see Data S1 for full WE‐ENACT survey). The WE‐ENACT survey has been refined over time based on cognitive testing and feedback from PCORI research partners, recommendations from PCORI's Advisory Panel on Patient Engagement and standard survey practices (eg retiring questions that have reached saturation, adding/modifying questions to capture new information). Refinements occurred prior to collection of the data presented in this paper. For the current study, partners responded to two open‐ended questions about (a) their main reason for wanting to contribute to the research project and (b) how their involvement in the project changed their lives (questions 2 and 6 in the WE‐ENACT survey document). Analyses of other survey questions are reported elsewhere.^9^, ^15^

### 2.3 Sample

Because partners were nominated to complete the WE‐ENACT annually, and participation was voluntary, partners could respond at different timepoints or multiple times during the data collection period (ie at project year 1, 2, and/or 3). A total of 798 partners were invited to take the survey at least one time; of those, 468 partners responded at least one time for a response rate of 59%. To represent different aspects of the patient experience, this study focused on responses from partners who identified as patients, family caregivers and representatives of patient and caregiver advocacy and community‐based organizations; we excluded responses from other types of stakeholders (eg clinicians, health systems, payers, policymakers). The final analytic sample included 396 responses from 255 PCORI research partners who provided data relevant to the analysis at one or more timepoints (see Data S2 for sample flow chart). Nearly half the partners in the final sample (n = 117) responded to the survey at multiple timepoints. Responses were completed at the end of project year 1 (n = 116), year 2 (n = 164) and year 3 (n = 116).

### 2.4 Analysis

We conducted descriptive statistics to examine characteristics of partners in the sample and the projects they represent. For the primary analysis, we developed a codebook to capture concepts reflected in the open‐ended responses to each of the two survey questions of interest in this study, resulting in a set of codes for each question. The codebook incorporated (a) concepts developed during previous examinations of WE‐ENACT data (eg ‘desire to help others', ‘gained new knowledge') and (b) concepts developed by analysing a sample of responses to each of the two questions (eg ‘belief in need for patient representation in research', ‘interest in topic area'). Because responses tended to be clear and brief, generally ranging from 30 to 60 words long, coding was conducted by a single analyst. During coding, if a response to one survey question included information relevant to the other survey question, the analyst applied the relevant codes to capture cross‐cutting content. After coding was completed, a team of three analysts conducted a thematic analysis of code reports, using a variety of well‐established techniques to draw conclusions from the data, such as identifying patterns, assessing the plausibility of findings and noting relationships among patterns.^22^ The research team then iteratively reviewed the themes to combine similar and overlapping items into the final themes. We examined whether frequency of themes varied by project year or previous experience as a partner on a research project but observed no notable differences, so results are presented aggregated across these characteristics. This qualitative analysis was carried out by a team of professional researchers. Patient and caregiver partners who participated in this study were not involved in analysing the data. However, as noted earlier, members of PCORI's Advisory Panel on Patient Engagement advised the research team on our analytic approach and contributed to reviewing and interpreting the qualitative themes.

## 3 RESULTS

Overall, most of the 255 partners in the sample were female, White and had high levels of educational attainment (almost 70% reported having a college or postgraduate degree) (Table 1). Partners represented 139 different projects, with one to nine partners responding per project (mean ± SD = 1.8 ± 1.2 partners per project). Projects in the sample address a range of health conditions and topics, including cancer, mental and behavioural health, and rare diseases, and used a variety of approaches to engage partners, such as advisory panels, focus groups and patient co‐investigators.^10^

**Table 1:** Characteristics of partners and projects in the study sample

Partner characteristics (N = 255)	N	%
Primary community represented (n, %)		
Patient/consumer	116	45%
Caregiver/family member of patient	51	20%
Patient, consumer or caregiver advocacy organization	41	16%
Community‐based organization	35	14%
Other^a^	12	5%
Age (mean ± SD in years)	55 ± 14	—
Gender (n, %)		
Female	174	68%
Male	62	24%
Another gender	1	<1%
Missing	18	7%
Race and ethnicity (n, %)		
American Indian/Alaska Native	3	1%
Asian	4	2%
Black or African American	33	13%
Native Hawaiian or other Pacific Islander	2	<1%
White	179	70%
Other	17	7%
Missing	17	7%
Ethnicity: Hispanic/Latino (n,% yes)	22	9%
Education (n, %)		
Less than high school	2	<1%
High school graduate or GED	7	3%
After high school training other than college (vocational or technical)	6	2%
Some college	47	18%
College graduate	74	29%
Postgraduate	100	39%
Missing	19	7%
Previous experience as a partner on a research project (n, % yes)	63	25%
Previously worked with current researchers (n, % yes)	53	21%

Project characteristics (N = 139 projects)	N	%
Primary health condition studied		
Cancer	20	14%
Mental/behavioural health	17	12%
Rare diseases	10	7%
Cardiovascular diseases	9	6%
Neurological disorders	9	6%
Nutritional and metabolic disorders	9	6%
All other health conditions	37	27%
Research with no specific health condition studied^b^	28	20%
PCORI research priority area		
Assessment of prevention, diagnosis and treatment options	47	34%
Improving health‐care systems	28	20%
Addressing disparities	24	17%
Accelerating patient‐centered outcomes research methods	22	16%
Communication and dissemination research	18	13%

a Includes partners who self‐identify as patient or family advocates, community advisors, peer group facilitators or as having multiple roles (eg both a patient and a caregiver).

b Includes studies focused on improving methods for patient‐centered outcomes research and studies that apply to a range of health conditions.

### 3.1 Motivations for engaging in research

When describing why they engaged in a particular research project, partners cited connections to their personal experiences living with or caring for someone with a particular health condition, working with people who have or are at risk of a condition, being part of a community affected by a condition, or participating in previous research. Five main reasons, or motivations, for engaging emerged (Table 2), ranging from improving people's lives to having a voice in research to learning more about a specific health topic. Patients, caregivers and representatives of advocacy and community‐based organizations expressed each of the five themes.

**Table 2:** Partners' motivations for engaging in research

Motivation themes	Exemplar responses
WE‐ENACT question: What is the main reason you want to contribute to this research project?	
Improving people's lives or health‐care experiences	‘When my mom had her stroke I was the decision maker. I will never forget how stressful that was. I want to help make the entire process as easy as it can possibly be for both the family member and the patient'. (*Caregiver/family member of patient)*
Addressing a gap for underserved communities	‘Because the research in the Latino community affected by cancer is insufficient and through research projects we will enhance the quality of life of future generations'. *(Representative of community‐based organization)*
Believing in the value of the health intervention	‘I see home‐based programs as a way people can understand their conditions and see the benefits of taking responsibility for their health'. (*Patient*)
Representing a perspective and having a voice in research	‘The main reason I wanted to contribute to this research project is to share my knowledge and life experiences so that others, in higher learning and the professional world, could understand that I have something of value to add to research, as well'. (*Representative of advocacy organization*)
Fulfilling a desire to learn	‘I had neuropathy, and I stopped taking my medications because it was doing other things to me. So, when I had the opportunity to learn more about it and that other patients were going through the same thing…I thought that was very interesting. I had questions about my medications and the impact it was having on other people'. (*Patient*)

#### 3.1.1 Improving people's lives or health‐care experiences

Partners reported wanting to help improve people's lives and patient outcomes by addressing such issues as survival, quality of life, access to care and prevention of health problems. For example, one patient partner from a project about serious mental illness said, ‘I want to contribute to this research project because I want to help improve the quality of life of others in need and I believe research is the best mechanism to do so'. Similarly, partners wanted to improve health‐care experiences and patient‐provider communication. Some highlighted motivations related to patient education and self‐management, such as increasing awareness of a condition or improving information for decision making.

#### 3.1.2 Addressing a gap for underserved communities

Partners noted that the population or condition of focus in the research project was understudied, underserved, poorly understood or had an unmet need. Examples of underserved populations included Latina, Black male, elderly, rural and native populations, as well as those with less access to specific types of health care. For example, one caregiver partner participating in a project examining telehealth services stated, ‘This project will fill a need of families in remote parts of the country, and someday the world, by connecting them with current up‐to‐date information that will help them support their children'.

#### 3.1.3 Believing in the value of the health intervention

Partners expressed a belief that the studied health intervention or treatment would help patients and families, and some were motivated by prior positive experience with the intervention. For instance, a partner identifying as an advocate for patients, families and children explained, ‘I believe in early intervention of [physical therapy/occupational therapy] as a significant factor in the success rate of patients with traumatic brain injury'.

#### 3.1.4 Representing a perspective and having a voice in research

Partners indicated the importance of people with their backgrounds or experiences participating in ‘research that affects their lives'. One patient partner stated, ‘Patients have a unique perspective on healthcare and can provide important information for improving healthcare'. Partners also characterized this as ‘having a voice' in research. Some expressed that all perspectives, such as patient, caregiver and clinician, should be represented in research. For instance, one partner representing a community‐based organization said, ‘This project places research partners at the table with researchers as co‐creators of knowledge and research'.

#### 3.1.5 Fulfilling a desire to learn

Partners expressed personal, professional or scientific curiosity and desire to learn about the study topic. Patient partners and caregivers typically wanted to learn more about their condition or about research that addressed issues relevant to their care. For example, a partner from an advocacy organization wrote about a desire to ‘learn how well [the state] is doing in addressing the healthcare needs of [patients with disabilities] and where improvements are needed'.

#### 3.1.6 Less commonly reported motivations

Another reported motivation was the desire to work with a specific investigator whose work or project was already known to the partner. Additionally, one caregiver partner mentioned more practical reasons for contributing: ‘If I could help any. It gave me an hour or so away from my wife, who has dementia and Parkinson's. Plus, they paid me $150'.

In summary, patients and caregivers in our study engaged as research partners because they sought to improve the lives of others and to support health‐care interventions that work for patients, including for underserved populations, as well as to have their perspective heard and to educate themselves about research and health topics. Notably, partners' motivations were primarily oriented towards benefiting others.

### 3.2 Changes in partners' lives from engagement

Five themes emerged from partners' descriptions of how engaging in the research project changed their lives, ranging from feeling they made a positive difference for patients to improving their personal health to developing skills and professional opportunities (Table 3). Patients, caregivers and representatives of advocacy and community‐based organizations expressed each of the five themes.

**Table 3:** Changes in partners' lives due to engaging in research

Change themes	Exemplar responses
WE‐ENACT question: Has your involvement in the project changed your life in any way? This might include things like building new relationships, better managing your health or finding new work opportunities. If so, please share.	
Making a difference	‘It is also a good feeling to know that our Board has helped to improve the treatment of other geriatric cancer patients'. *(Caregiver/family member of patient)*
Building new or better relationships	‘I have developed important relationships and feel tremendous loyalty to the entire team and its goals'. (*Representative of advocacy organization*)
Developing greater knowledge and appreciation of research	‘It's been personally helpful to improve my knowledge about how research works'. *(Representative of community‐based organization)*
Improving personal health and health care	‘It taught me how to be more adherent to my medicine. I got healthier and more consistent with my medicine while teaching my peers to be healthy…It's had a great impact on myself'. *(Patient)*
Developing skills and professional opportunities	‘[It] allowed me to create a patient presence at conferences. Supported me in taking the steps to get into medical school'. (*Patient*)

#### 3.2.1 Making a difference

Partners discussed feeling that their contributions helped improve health care or the lives of patients. Some partners focused on making a broad difference in how health‐care decision making occurs. One patient shared, ‘My involvement in the project has given me a sense of contributing to a meaningful cause after my retirement.'

Others indicated that their experience gave them hope for the future. For example, one partner representing an advocacy organization wrote, ‘Yes, it has given me hope that ‘the unheard' have had their say and will not only be listened to but changes will be implemented in the future of our state's healthcare'.

#### 3.2.2 Building new or better relationships

Partners indicated developing new relationships as a result of engaging in research. For example, partners described connecting with and learning from other patients and caregivers and drawing strength from that experience. One caregiver partner from a project about paediatric diabetes said, ‘The bond of understanding I have with the other involved parents is reassuring. Knowing that we all have the same struggles and knowing that we are helping to make some of these struggles easier for other families is very rewarding'.

Partners also noted that they and/or the organizations they represent developed new or better relations with researchers. For instance, one partner representing a community‐based organization said, ‘It has developed positive relationships between our non‐profit agency and the University researchers'.

#### 3.2.3 Developing greater knowledge and appreciation of research

Partners discussed gaining an improved understanding of research and wanting to continue being involved in research. Partners noted an increase in knowledge of research methods and the process of conducting a study, a better understanding of the research topic and its related issues, and participation in dissemination activities, such as conferences and interviews with news media.

Partners also reported having a positive experience as part of the research team. For example, one caregiver partner noted, ‘This is the first opportunity that I have had the privilege to be a part of where the researchers really wanted to know what families think and to listen to their real‐life stories'. Other partners expressed appreciation of the researchers they worked with, noting both personal and professional qualities they admired and that affected them; partners used words such as innovative, outstanding or dedicated. Some partners discussed obtaining and or pursuing new research opportunities, including follow‐on projects with the same research group and new opportunities identified while engaged on the project.

#### 3.2.4 Improving personal health and health care

Partners described improvements to their own health or health‐care habits. For example, partners noted learning new skills or mastering technology to manage their health, increasing medication adherence, visiting their clinician more often, improving their use of preventive care and asking more questions or seeking more information about their care. One patient partner said, ‘I feel that it helped me to be a better advocate for myself as a patient, and it helped me to better communicate with my team of doctors'. Similarly, a partner from an advocacy organization engaged in a project about multiple chronic conditions shared, ‘My health is better. I eat healthier and take care better care of myself, physically and mentally, to prevent some of the diseases I have encountered with the participants I serve'. Partners also reported gaining a better understanding of patients, clinicians and the health‐care system.

#### 3.2.5 Developing skills and professional opportunities

Partners described several ways their experience led to self‐improvement, such as gaining confidence or becoming a better teammate. Others, especially those representing community‐based organizations, indicated that they became better at their primary role or job by developing knowledge or skills that were immediately useful or gaining understanding and empathy for patients. For instance, one partner representing a community‐based organization stated, ‘I've learned about how such tools could be implemented and used to enrich patients' lives. My own work has become more patient‐centered and I've improved my ability to work as part of a team'.

Partners also discussed obtaining or pursuing a new job or responsibilities, such as a seat on an organizational board, going to medical school or changing to a job more closely related to their role on the research project. As one partner from an advocacy organization wrote, ‘This project has helped me to expand my thinking…to redefine who I am, and work towards the goal of making it my career'.

#### 3.2.6 No changes

Some partners reported that engaging in research had not led to any changes in their lives. While most of these partners did not expand on their answer, some noted they still had a positive experience as a research partner. A few partners stated that the project was in an early stage or that they were not involved enough to experience any influences. For example, one patient partner wrote, ‘I am only working on the project approximately once a month so nothing has greatly changed my life'.

Although a small minority of partners did not believe that engaging in research changed their lives in any way, most partners described at least one change. Partners experienced a sense of contributing to others' lives, new relationships, enhanced knowledge and enthusiasm for research, improved health, and new skills or professional opportunities. Notably, partners did not describe any negative changes in response to this survey question.

While partners' motivations for engaging in research were oriented primarily towards benefiting others, their perceptions of changes in their lives reflect both benefits to others and multiple benefits to themselves. Our study did not examine how individual partners' motivations for engaging in research relate to their perceptions of changes in their lives. Collectively, however, the findings suggest that partners do experience benefits that align with commonly reported motivations for engaging. For example, many partners were motivated by a desire to help others, and many reported feeling they made a positive difference in patients' lives.

## 4 DISCUSSION

This study provides new information about why US patients, caregivers and representatives of patient and caregiver advocacy and community‐based organizations engage in health research and how their experiences as research partners affect their lives. By identifying what partners value and how they may benefit from their research partnerships, these findings point to possible ways of attracting and maintaining research partnerships and add to the evidence base about the value of engagement.

Partners in our study described several of the same motivations for and benefits of research engagement as partners in prior studies conducted primarily in the UK.^11^, ^16^, ^17^, ^23^, ^24^ Our study also identified two unique motivations for engaging in research: belief in the value of a particular health intervention and desire to represent an underserved population. These motivations may reflect PCORI's focus on CER studies comparing different interventions or health‐care approaches among real‐world populations. Overall, the consistency of motivators and benefits of engagement across different populations, health care and research contexts, and research methods bolsters our confidence in this small but growing body of research and suggests there are many commonalities in partners' experiences with engagement.

When asked how engaging in research changed their lives, no partners in this study talked about negative changes. However, as reported elsewhere, when surveyed about challenging aspects of engaging, PCORI research partners described such experiences as difficulty managing competing demands, unmet expectations about project progress and insufficient communication about how their input was used.^15^ Other literature has described similar challenges of patient engagement in research.^11^, ^13^, ^16^ However, our study adds to the evidence showing that partners also experience many benefits from engaging in research. For example, patient partners reported making improvements to their own health management and learning to better utilize the health‐care system, suggesting that engagement in research may be related to engagement or activation for health care, and perhaps ultimately, better individual health outcomes.^25^

### 4.1 Implications for engagement policy and practice

In addition to improving partners' lives, benefits of engaging in research may promote more collaborative and sustainable research partnerships by encouraging partners to stay actively engaged in a project or to pursue additional engagement opportunities. Policymakers, research funders and research institutions considering whether or how to create policies and allocate resources for patient and stakeholder engagement in research should be aware of the growing evidence that engagement benefits not only research but also people engaged as partners. More concretely, our study's findings suggest possible strategies for researchers and research partners to facilitate engagement at key junctures, including initiating partnerships, developing engagement plans, and fostering and maintaining rewarding partnerships over time.

First, researchers can draw on the motivations and benefits identified in our study and others^11^, ^16^, ^19^, ^23^, ^26^ to communicate with potential partners about engagement opportunities and to develop engagement plans. Although researchers share some motivations with patients and caregivers, such as helping patients and improving care, researchers must recognize that some of their reasons for engagement, such as robust study enrolment and retention, may be lower priorities for partners.^24^, ^27^ Therefore, researchers should work with partners to plan roles that align with partners' motivations. For example, researchers may have more success attracting patient and caregiver partners if they talk with potential partners about how their involvement in the project can potentially help patients and how partners may personally benefit. For potential research partners, who have competing personal and professional demands, knowing how an opportunity to engage in research aligns with their motivations and how they might benefit can empower them to make informed decisions about whether and how much to engage in a project.

Our findings also point to some tangible ways for research teams to foster and maintain rewarding partnerships by attending to partners' motivations and experiences throughout the research process. To be responsive to partners' desires to make a difference, researchers and partners should establish regular communication about how partner input contributes to more patient‐centered research. At the same time, partners may sometimes offer input that cannot be implemented feasibly given real‐world constraints of study scope, methodological rigour or resources, and it typically takes several years to conduct a study and implement the findings. Thus, researchers and partners should also consider how to establish shared, realistic expectations about the research process and the pace of impact on clinical practice, so that partners who want to make a difference do not lose motivation to stay engaged in the study or engage in future studies. More broadly, researchers and partners should continue to discuss partners' goals and experiences with engagement throughout the project, not just when initiating partnerships, to ensure that partners' roles on the project continue to be rewarding. Lastly, although partners in our study described primarily altruistic, and not financial, motivations for engaging in research, fair and appropriate compensation tailored to partners' needs is a foundational part of equitable and trustworthy partnerships.^28^

### 4.2 Limitations and future research

This study has some notable limitations. Partners included in the study were selected by the project's principal investigator to receive a survey invitation, participation was voluntary, and the survey was administered in English only. Investigators may have nominated partners who had more positive experiences. Although our study's sample is more diverse than prior research in this area,^16^ the majority of partners in our sample were non‐Hispanic, White, female and had high levels of educational attainment. We are unable to determine whether partners in this study are representative of PCORI partners overall. We also could not adequately explore potential differences in responses according to partners' race, ethnicity or education. Future studies should be designed to ensure a more diverse sample of partners, with particular attention to inclusion of groups with a history of underrepresentation in research.

The survey question asking partners how engagement changed their lives included examples that could have steered some partners towards certain responses (see Table 3). However, partners were able to meaningfully elaborate on their experiences, and many partners described additional changes beyond the given examples.

Partners in the sample vary in terms of how many times and when in the project (eg year 1, 2, and/or 3) they responded to the survey. The timing or frequency of data collection may have influenced partners' responses, particularly because partners may perceive more changes in their lives later in a project. However, the themes we observed in partners' responses about their motivations for engaging and changes in their lives did not differ by project year.

Finally, our findings could be enhanced by additional information about aspects of partners' involvement in research that may have a bearing on their motivations and perceptions of changes in their lives, such as how they initially got involved in the project, whether they received training, and how long they engaged as partners on the project.

Despite these limitations, this study provides valuable insights into the experiences of patients, caregivers and representatives of advocacy and community‐based organizations engaged in health research as part of a US movement towards more patient‐centered research. To our knowledge, no research funder other than PCORI regularly and systematically collects information from research partners about their experiences. To inform more targeted approaches for developing partnerships with diverse patients and stakeholders, future research should examine motivations for and benefits from engagement among patient groups with a history of underrepresentation in research and seek to understand differences among various patient subpopulations, such as those with different health conditions and levels of involvement in research. It is also important to consider the motivations and experiences of other stakeholders, such as clinicians, representatives of health systems, payers and policymakers, who need relevant, trustworthy evidence and should also be key partners in developing that evidence. Additionally, to fully understand how the experience of engagement contributes to the culture of the research community, future studies should also further examine how engagement affects researchers,^11^ their research programmes and their institutions.

## 5 CONCLUSIONS

PCORI's main objective is to fund research that produces more useful, relevant and patient‐centered evidence to guide health‐care decisions of patients, caregivers, clinicians and other stakeholders. Engaging stakeholders, especially patients, as partners in all aspects of the research process is a key strategy to achieve this objective. Although engagement in research can be time‐consuming and challenging, growing evidence shows that engagement benefits not only the research itself but also people involved as partners, adding to the case for making engagement more widespread. As the evidence base for research engagement continues to grow, more organizations, including funders, academic institutions and health‐care systems, likely will consider implementing or refining policies and resources to support engagement, and opportunities for patients to engage in research are likely to grow. By illuminating why patients and caregivers engage as partners in health research and what they get out of the experience, our findings can guide researchers in designing strategies to attract partners and foster mutually beneficial and sustainable research partnerships.

## CONFLICT OF INTEREST

The authors have no conflicts of interest to disclose.

## Data Availability

Research data are not shared due to confidentiality and parameters of the consent obtained. Please contact the corresponding author for more information.
