# Supplementary material for: What motivates patients and caregivers to engage in health research and how engagement affects their lives: Qualitative survey findings
Source: Health Expect. 2019 Dec 4;23(2):328–36. doi: 10.1111/hex.12979 (PMC7104645; doi:10.1111/hex.12979)
Supplement: Supplementary file 1 [file HEX-23-328-s001.pdf]

Supporting Information 1: WE-ENACT Survey.

Ways of Engaging- ENgagement ACtivity Tool (WE-ENACT) - Patients and Stakeholders 3.0 Item Pool

This inventory includes a pool of items to describe engagement from the patient and stakeholder point of view, from which relevant items of interested can be selected. This tool is designed to be flexible to apply to multiple time points in the project (annual intervals) by changing the time reference in each item.

Ways of Engaging- Engagement ACtivity Tool (WE-ENACT)

This survey is about the role of patients and other health care stakeholders (for example, family caregivers, doctors, hospital leaders, and insurance companies) in research projects. These questions will ask about your involvement in the PCORI-funded research project, including:

- What you did and how it made a difference
- How the research team worked together

We want to learn about your experiences with this PCORI project. Specifically, we are interested in your experiences *<insert time>.*

1. Patients and stakeholders can contribute to research projects in many ways. This could include:

   - Making sure researchers know what kind of information is important to patients;
   - Deciding what the study should be about;
   - Deciding who to include in the study;
   - Choosing what outcomes the study will measure;
   - Tracking study progress;
   - Sharing study findings

   We call contributing to research projects in ways like this "being a research partner". Were you a research partner on this project *<insert time>?*

   ☐Yes
   ☐No → **go to Q23**

2. **[For first time survey respondents]** What is the main reason you want to contribute to this research project?

3. People can be involved in many parts of a research project. Please select all of the parts you have been involved in *<insert time>.*

☐ Helping researchers understand what information patients and stakeholders need
☐ Choosing the research topics or the medical questions to study
☐ Choosing or designing the program or treatments being compared
☐ Choosing the outcomes that matter to patients (what the study will measure)
☐ Training research staff on how to recruit and work with patients
☐ Finding patients to participate in the study or making it easier for them participate
☐ Collecting data from study participants
☐ Analyzing or reviewing results
☐ Explaining or applying results to real world settings
☐ Sharing study findings with those who can use them
☐ Other part of the project– *Please describe*:

4. **[For each part of the research project selected in Q3] For each part of the research project below, describe what you did and how it made a difference.** You can fill in as much or as little of this table as you like.

(Continued on next page)

	What did you do?	***How did this make a difference?***
• ***Example: Choosing the research topics or the medical questions to study***	I explained to others on the research team what research questions matter most to patients	The research questions were changed to include the questions important to patients
• ***Helping researchers understand what information patients and stakeholders need***		
• ***Choosing the research topics or the medical questions to study***		
• ***Choosing or designing the program or treatments being compared***		

For information about the WE-ENACT or to inquire about its use please contact surveys@pcori.org.

08.01.2016

• ***Choosing the outcomes that matter to patients (what the study will measure)***		
• ***Training research staff on how to recruit and work with patients***		
• ***Finding patients to participate in the study or making it easier for them to participate***		
• ***Collecting data from study participants***		
• ***Analyzing or reviewing results***		
• ***Explaining or applying results to real world settings***		
• ***Sharing study findings with those who can use them***		
• ***Other (as described)***		

5. **[For approaches to engagement selected in Q3]** How much influence did you have <*insert time*> on each part of the project listed below?

	Amount of Influence				
	None	*A small amount*	*A moderate amount*	*A great deal*	*I don't know whether I influenced this part*
a. Helping researchers understand what information patients and stakeholders need	☐	☐	☐	☐	☐
b. Choosing the research topics or the medical questions to study	☐	☐	☐	☐	☐
c. Choosing or designing the program or treatments being compared	☐	☐	☐	☐	☐
d. Choosing outcomes that matter to patients (what the study will measure)	☐	☐	☐	☐	☐
e. Training research staff on how to recruit and work with patients	☐	☐	☐	☐	☐

f. Finding patients to participate in the study or making it easier for them participate	☐	☐	☐	☐	☐
g. Collecting data from study participants	☐	☐	☐	☐	☐
h. Analyzing or reviewing results	☐	☐	☐	☐	☐
i. Explaining or applying results to real world settings	☐	☐	☐	☐	☐
j. Sharing study findings with those who can use them	☐	☐	☐	☐	☐
k. <Other, as described in Q3>	☐	☐	☐	☐	☐
l. The way the research team and stakeholder partners work together	☐	☐	☐	☐	☐

6. Has your involvement in the project <*insert time*> changed your life in any way? This might include things like building new relationships, better managing your health, or finding new work opportunities. If so, please share.

7. Please select the one that best reflects your role for this research project.

   ☐ Patient/Consumer
   ☐ Caregiver/Family Member of Patient
   ☐ Patient, Consumer, or Caregiver Advocacy Organization
   ☐ Community-Based Organization (CBO)
   ☐ Clinician
   ☐ Clinic/Hospital/Health System
   ☐ Purchaser (small or large employer)
   ☐ Payer (public or private insurance)
   ☐ Life Sciences Industry
   ☐ Policy Maker (government official)
   ☐ Training Institution (non-research health professions educator)
   ☐ Subject Matter Expert
   ☐ Other – *Please describe* ______________________________

For information about the WE-ENACT or to inquire about its use please contact surveys@pcori.org.

8. *<insert time>,* how much did you feel trust, honesty, transparency, shared-learning, and give-and-take relationships while working on this project?

   ☐ Not at all
   ☐ A little bit
   ☐ Somewhat
   ☐ A great deal

9. **[If Q8 = Somewhat OR A great deal]** Please provide an example of how you experienced trust, honesty, transparency, shared-learning, or give-and-take relationships *<insert time>.*

10. **Optional:** Please provide any additional thoughts about the way researchers, patients, and/or other stakeholders worked together on this research project.

11. Sometimes there are challenges when researchers, patients, and other stakeholders work together. These might include finding a convenient time to meet or communicating clearly with each other. What have been the biggest challenges for you on this research project *<insert time>*?

12. How prepared did you feel to contribute to this research project *<insert time>*?

   ☐ Not at all
   ☐ A little bit
   ☐ Somewhat
   ☐ A great deal

13. **[If Q12 = Somewhat OR A great deal]** What training, support, or past experiences helped you feel prepared?

14. **Optional:** What additional training or support would you like to have received?

15. **Optional:** Please share anything else that helped you contribute to this research project *<insert time>*. For example, this may include things you did to ensure your view was heard or things the researchers did to ensure everyone was included.

For information about the WE-ENACT or to inquire about its use please contact surveys@pcori.org.

16. Based on your experience with this research project, what would you suggest be done to help others contribute as research partners?

17. What aspects of working on this project did you **not** like?

18. If the opportunity arose, would you be interested in working as a research partner on another research study?

☐ Yes
☐ No

19. **[If Q18=No]** Why would you not be interested in working as a research partner on another study?

20. **[For first time survey respondents]** Had you worked with any of these researchers before this project?

☐ Yes
☐ No

21. **[For first time survey respondents]** **[If Q20= Yes]** Altogether, how long have you worked with these researchers?

_____Years _____ Months

22. **[For first time survey respondents]** Prior to working on **this** project, had you contributed to a research study as a research partner?

☐ Yes
☐ No

For information about the WE-ENACT or to inquire about its use please contact surveys@pcori.org.

08.01.2016

Demographic Questions
[For first time survey respondents] Now we have a few questions about you. This information helps us understand who has responded to the survey. **These questions are optional.**

23. Please select your gender
    - ☐ Male
    - ☐ Female
    - ☐ Other (please describe)

24. What is the highest level of formal education you completed?
    - ☐ Less than high school
    - ☐ High school graduate or GED
    - ☐ Post high school training other than college (vocational or technical)
    - ☐ Some college
    - ☐ College graduate
    - ☐ Postgraduate

25. Which best describes your race?
    - ☐ American Indian/ Alaska Native
    - ☐ Asian
    - ☐ Native Hawaiian or other Pacific Islander
    - ☐ Black or African American
    - ☐ White
    - ☐ Other

26. Do you consider yourself to be Hispanic or Latino?
    - ☐ Yes
    - ☐ No

27. What is your age? ___

28. Your views are very valuable to us. We welcome any additional thoughts, information, or stories about your experience with this research project.

For information about the WE-ENACT or to inquire about its use please contact surveys@pcori.org.

08.01.2016
