# Supplementary figures and images for: What motivates patients and caregivers to engage in health research and how engagement affects their lives: Qualitative survey findings
Source: Health Expect. 2019 Dec 4;23(2):328–36. doi: 10.1111/hex.12979 (PMC7104645; doi:10.1111/hex.12979)

## Supporting Information 2: Study Sample Flow Chart

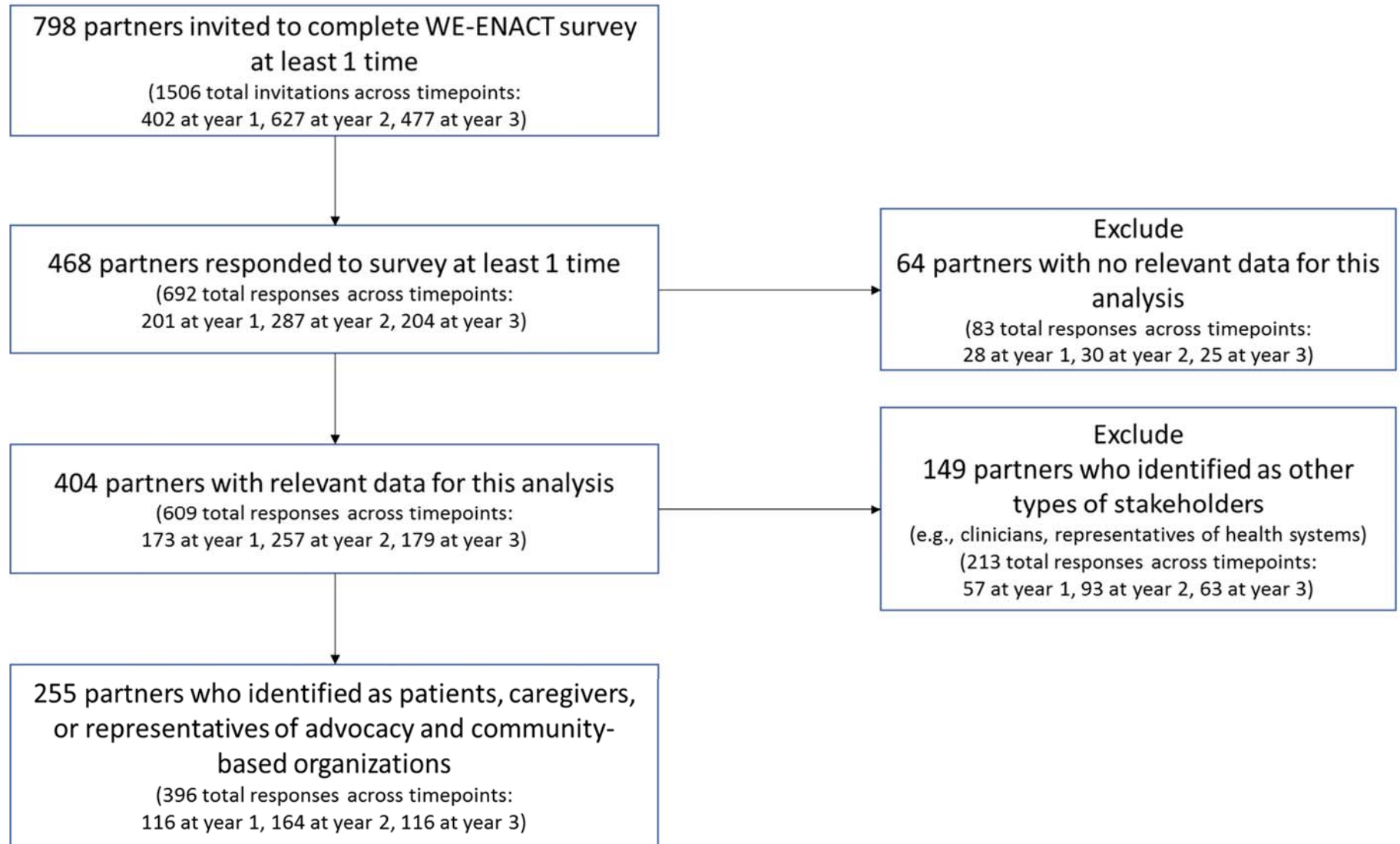

Supplement: Supplementary file 2 [file HEX-23-328-s002.pdf]
